# Different co-sensitizations could determine different risk assessment in peach allergy? Evaluation of an anaphylactic biomarker in Pru p 3 positive patients

**DOI:** 10.1186/s12948-015-0035-7

**Published:** 2015-12-02

**Authors:** Carina Gabriela Uasuf, Danilo Villalta, Maria Elisabetta Conte, Caterina Di Sano, Maria Barrale, Vincenzo Cantisano, Elisabetta Pace, Mark Gjomarkaj, Sebastiano Gangemi, Ignazio Brusca

**Affiliations:** Allergy Diseases Center “Prof. G. Bonsignore”, Institute of Biomedicine and Molecular Immunology “A. Monroy”(IBIM)-National Research Council (CNR), Palermo, Italy; Allergy and Clinical Immunology Unit, A.O “S. Maria degli Angeli di Pordenone”, Pordenone, Italy; Clinical Pathology, Allergy Unit, Buccheri La Ferla Hospital, Palermo, Italy; School and Division of Allergy and Clinical Immunology, Department of Clinical and Experimental Medicine, University of Messina, Messina, Italy

**Keywords:** nsLTP, Peach allergy, sIgE to Pru p 3, Anaphylactic biomarker, Risk assessment, Co-sensitization, Molecular allergy based diagnosis

## Abstract

**Background:**

In Italy, the nsLTP (Pru p 3) has been identified as the most frequent cause of food allergy and anaphylaxis. In order to estimate the risk assessment in peach allergy, we investigated the presence of correlations between the levels of sIgE to Pru p 3 with the severity of the clinical symptoms in two Pru p 3 positive populations from two different areas of Italy.

**Methods:**

133 consecutively Pru p 3 positive patients were recruited from South Italy, where the prevalence of PR-10 and profilin sensitization is low, and from North-East Italy, where the sensitization to pathogenesis related protein -10 (PR-10) and profilin is higher. Skin prick test (SPT) to peach extract and sIgE to peach panallergens were performed.

**Results:**

All 133 patients were positive to SPT to peach extract and to sIgE to Pru p 3. The North-East population was simultaneously positive to Pru p 1 (42.8 %) and Pru p 4 (12.7 %), while no Southern patients were positive to PR-10 or to profilin. A significant difference in the levels of sIgE to Pru p 3 was found only in South Italy Pru p 3 + patients vs. asymptomatic patients (p = 0.01) and in mild reactions vs. severe reactions (p = 0.0008). In South Italy patients, it was also found a significant correlation between the severity of the clinical reaction and the levels of sIgE to Pru p 3 (p = 0.001).

**Conclusion:**

Level of sIgE to Pru p 3 indicates the possibility of development a severe food allergic reaction. Pru p 3 positive patients from different geographical areas and with different co-sensitizations to Pru p 1 and/or Pru p 4 could have a different risk assessment in peach allergy.

## Background

The possibility to predict, by in vitro approaches, the development of symptoms related to food allergy (FA) has been studied by different research groups and several data have been published focusing on this point. Interestingly, some results have demonstrated that the levels of specific IgE (sIgE) for food allergens can be considered a reliable marker of a positive “in vivo” challenge test. [1–3]. Currently, the risk assessment of FA can be predicted in a more accurate way since the development of the molecular allergy based diagnosis, although it’s still not possible to distinguish between patients who are asymptomatic from those symptomatic.

In FA, the molecular allergy based diagnosis has the peculiarity of identify different type of sensitizations depending on the geographical area. For example, it has been shown that the sensitization to the panallergen non-specific lipid transfer protein (nsLTP) increases from North Europe to the Mediterranean area, while the sensitization to the pathogenesis related protein-10 (PR-10) increases in the opposite direction. By contrast, the frequency of sensitization to profilin is almost invariable.

Several studies showed that Pru p 3, the peach nsLTP, is the more frequent cause of food-induced allergy and anaphylaxis in Italy [4–6]. The clinical manifestations of nsLTP sensitization range from those of minor entity, like contact itch or oral allergic syndrome (OAS), to anaphylactic shock. [7].

The sensitization to this heat and proteolytic digestion resistant protein is potentially very dangerous [8]. Cross reactions between peach nsLTP and nsLTP from other foods, have been described with other members of the Rosaceae family, seed, nuts, cereals, and other foods from plant origin [9–19].

The relationship between the levels of sIgE, the severity of the clinical reactions and the presence of symptoms with foods containing nsLTP different from peach, is presently controversial [20–23].

In consideration of the potential hazard of the sensitization to Pru p 3, it is evident how important is to identify a biomarker able to predict both the severity of an allergic reaction and the potential cross reactions with other nsLTP foods.

The aim of this study was to investigate a possible correlation between an anaphylactic biomarker, such as sIgE to Pru p 3, with the severity of symptoms in two Pru p 3 sensitized populations from different geographical areas of Italy. In addition, we performed the ROC curve analysis of the sIgE to Pru p 3 in order to identify patients at a higher risk of anaphylaxis and compare our results with the data published in literature.

## Methods

### Patients

133 consecutively allergic patients who attended the observation were recruited. A common selection criteria was used in all participating center: (a) clear history of an allergic reaction after being in contact with/or after the consumption of peach, (b) skin prick test (SPT) positive to peach extract, (c) positive sIgE to Pru p 3, (d) positive oral open food challenge or in case the challenge could not been performed, a well-documented severe allergy had been exhibited, e) patients positive to SPT to peach and to sIgE to Pru p 3 but asymptomatic. None of the patients were under oral corticosteroid or antihistamine.

All 71 patients who attended the outpatient Allergy Unit of C.N.R. and Buccheri La Ferla Hospital were born and live in Palermo (South Italy). All 62 patients who attended the outpatient Allergy Unit of Santa Maria degli Angeli Hospital were born and live in Pordenone (North-East, Italy).

Patients were divided into four groups based on severity of symptoms: asymptomatic, OAS, mild systemic reactions (Mueller classification grade 1–2), and severe systemic reactions (Mueller classification grade 3–4) (Table 1) [24].

**Table 1:** Demographic and clinical characteristics of sensitized peach patients from North-East and South Italy

	South Italy	North-East Italy
Patients (n)	71	62
Sex (f/m)	41/30	34/28
Age (mean, years)	25.7	38.03
Clinical manifestation (n)		
Asymptomatic	5	13
OAS	7	28
Mild reactions	40	17
Severe reactions	19 (7 anaphylactic shock)	4 (1 anaphylactic shock)

### Skin prick tests

SPTs to peach extract (ALK-Abello, Milan, Italy) were performed consecutively by the same investigator in the South population and by another investigator in the North-East population, on the volar surface of the forearm by using a standard l mm tip lancet, according to the recommendations of the European Academy of Allergy and Clinical Immunology group. Histamine hydrochloride (10 mg/mL) and saline (ALK-Abello, Milan, Italy), respectively, were used as positive and negative controls.

The SPT results were considered positive if the difference between the mean diameter of the wheal and the negative control was at least of 3 mm.

### sIgE dosage

sIgE anti-rPru p 1, anti-rPru p 3, and anti-rPru p 4 were consecutively determined using the ImmunoCAP system (Thermofisher/Phadia Diagnostics, Uppsala, Sweden). Results were expressed in kUA/L and a value >0.15 kUA/L was considered positive.

All serum samples were consecutively analysis by both Buccheri La Ferla and Maria degli Angeli laboratories. The calibration test was done when the ImmunoCAP 250 required. Every day, new reagents were used due to the high number of samples analysed by both laboratories. During every single session, two control curves and one quality control (given by Thermofisher/Phadia Diagnostics) were performed. In addition, measurements of sIgE to peach and to Pru p 3 were done using a “house control” (obtained from a serum pool frozen at −80 °C).

Both laboratories have been participated to external VEQ specific allergy programs: Buccheri La Ferla laboratory has been participated to the Sicilian Region CRQ and the laboratory from Pordenone, to the NEQAS UK.

To be sure the results were reproducible, all 133 serum samples were analysed by Buccheri La Ferla laboratory, during a single session, using the same batch of reagents.

### Oral food challenge

Patients with OAS reintroduced peaches under our supervision and experienced local symptoms of OAS. In view of the observational nature of the study and of the fear of several patients of a possibly severe adverse reaction, patients with systemic reactions were not challenged because of documented severe peach allergy [25].

The study was performed with the approval of the ethics committee of Policlinico—Giaccone Hospital—Palermo Italy (7/2013–12.06.2013) and was in agreement with the Helsinki Declaration. Written consent form was obtained from the patients.

### Statistical methods

The association between levels of sIgE to Pru p 3 in asymptomatic vs. symptomatic patients with local and systemic reactions were analyzed by Mann–Whitney test.

The ROC analysis was carried out to determine the optimal cut-off value of sIgE to Pru p 3 to distinguish asymptomatic from symptomatic patients both in South and North-East Italian populations.

Correlations between sIgE levels and symptom severity were assessed using Spearman rank correlation (rho). In addition, the odds ratio of sIgE levels to Pru p 3 was calculated to assess the risk of severe reactions. The latest test was not done in the North-East group due to the low number of patients with severe reactions.

Medcalc 11.4.4.0 was used for the statistical analyses. A *p* value of <0.05 was considered significant.

## Results

All South and North-East Italian patients were positive (mean diameter > 3 mm) to SPT to peach extract and to sIgE to Pru p 3. The sensitization to other panallergens differs according to the geographical area: the North-East population was simultaneously positive to Pru p 1 (42.8 %) and Pru p 4 (12.7 %), while no Southern patients were positive to PR-10 or to profilin (Table 2).

**Table 2:** Difference in sensitization to peach allergens between the North-East and the South Italian patients

	Pru p 1	Pru p 4	Pru p 3
Palermo n = 71 (%)	0*	0**	100
Pordenone n = 62 (%)	42.8	12.7	100

* p < 0.0001

** p = 0.005

**Fig. 1:**
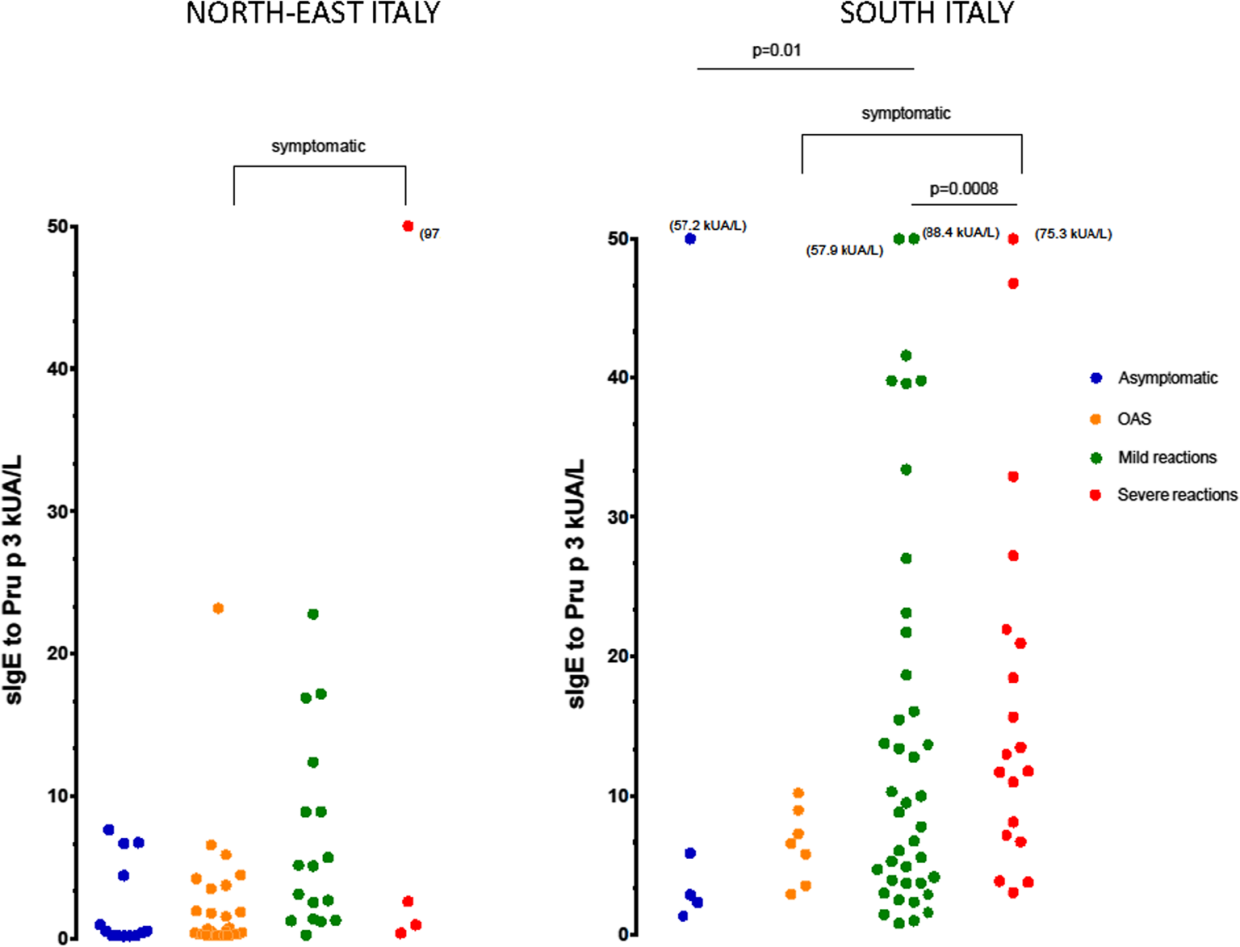
Levels of sIgE to Pru p 3 in peach allergic patients from South and North-East Italy according to the clinical reactions

The levels of sIgE to Pru p 3 from the two populations are shown in Fig. 1.

In the South population, it was found a significant difference in the levels of sIgE to Pru p 3 between all symptomatic patients (OAS, mild, and severe systemic reactions) vs. asymptomatic patients (p = 0.01), and between patients with mild systemic reactions compare with patients with severe systemic reactions (p = 0.0008). On the other hand, in the North-East population, not significant differences were found in the levels of sIgE to Pru p 3 between symptomatic (OAS, mild, and severe systemic reactions) vs. asymptomatic patients (p = 0.08) and between patients with mild systemic reactions compared with patients with severe systemic reactions (p = 0.09).

Interestingly, in the South population was found a correlation between the severity of the clinical reactions and the levels of sIgE to Pru p 3 (Spearman’s test = 0.366; p = 0.001). Unfortunately, in the North-East population, not correlation test was done due to the low number of patients with severe allergic reactions.

The ROC curve analysis from all South Pru p 3 positive patients identified a value of sIgE to

**Fig. 2:**
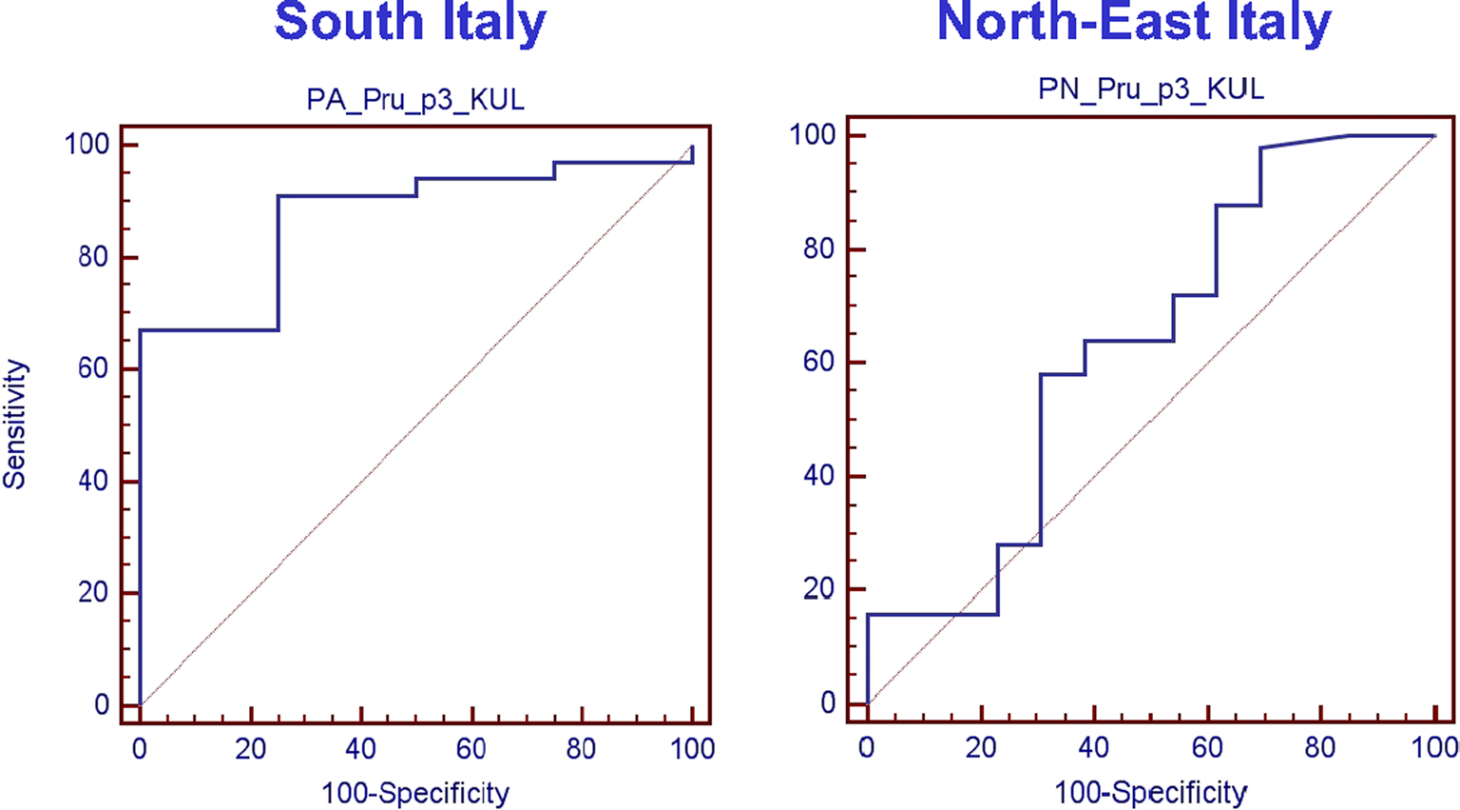
ROC curves for sIgE to Pru p 3 values in all peach allergic patients from South and North-East Italy

Pru p 3 of 2.87 kUA/L as the best value discriminating asymptomatic from symptomatic (OAS, mild, and severe systemic reactions) patients [sensitivity 91.04 % (95 % CI 81.5–96.6); specificity 75 % (95 % CI 19.4–99.4); LR + 3.64 (95 % CI 2.1–6.4); Fig. 2].

A value of sIgE to Pru p 3 of 5.87 kUA/L has a sensitivity of 67.16 % (95 % CI 54.6–78.2) and a specificity of 100 % (95 % CI 39.8–100.0).

The ROC curve analysis from all North-East Pru p 3 positive patients identified a value of sIgE to Pru p 3 of 2.68 kUA/L as the best value that discriminated asymptomatic from symptomatic (OAS, mild, and severe systemic reactions) patients [sensitivity 36.0 % (95 % CI 22.9–50.8); specificity 69.2 % (95 % CI 38.6–90.9); LR + 1.17 (95 % CI 0.7–2.0); Fig. 2].

**Fig. 3:**
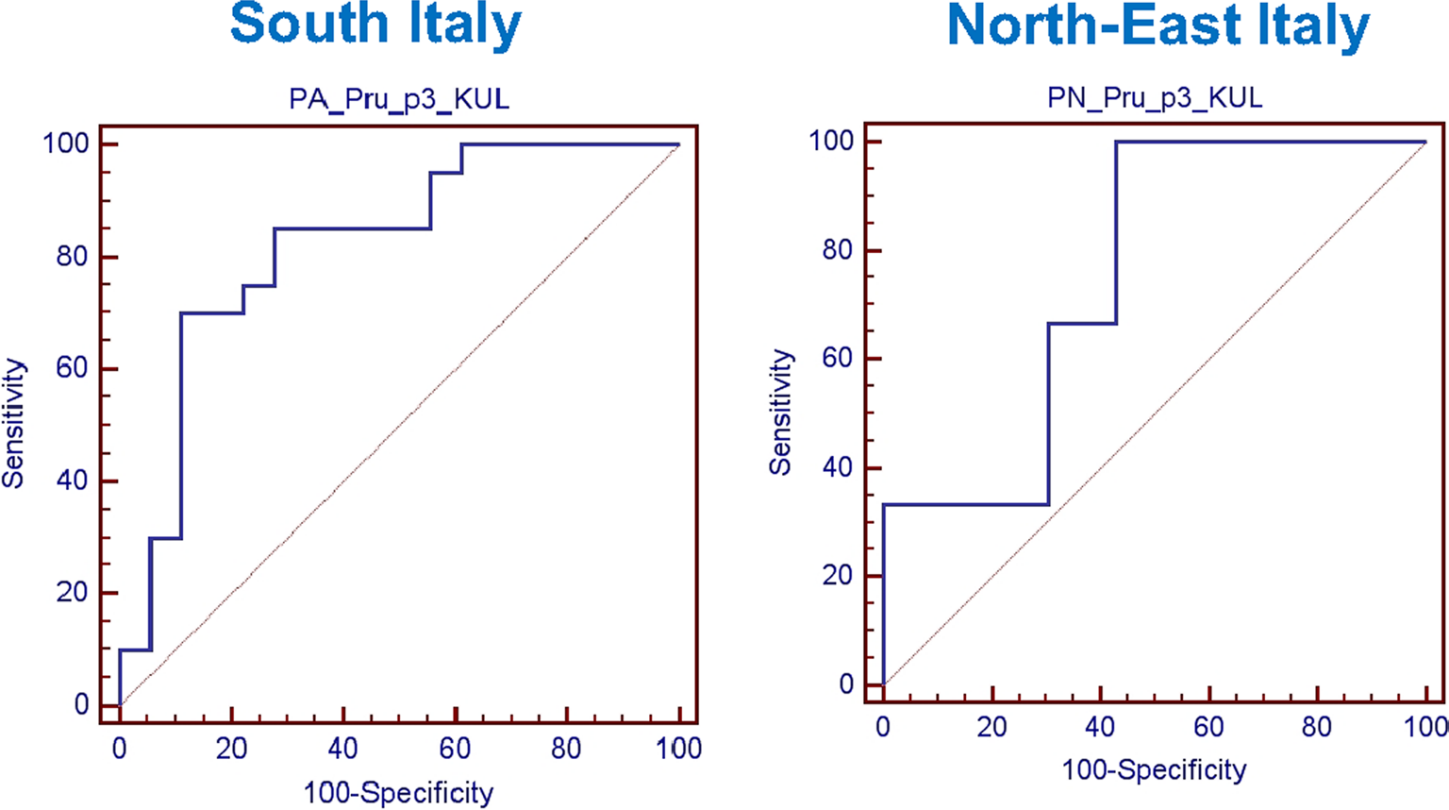
ROC curve for sIgE to Pru p 3 values in patients with severe allergic reactions from South and North-East Italy

In the South group, the value of sIgE to Pru p 3 of 10.2 kUA/L was the best value that discriminated severe reactions from other clinical reactions [sensitivity 70.0 % (95 % CI 45.7–88.1); specificity 88.9 % (95 % CI 65.3–98.6); LR + 6.30 (95 % CI 4.5–8.8)]. Due to the low number of severe reactions in the North-East population, it was not possible identify a value of sIgE to Pru p 3 able to discriminate severe reactions from other clinical reactions (Fig. 3).

The area under curve (AUC) of symptomatic (OAS, mild, and severe systemic reactions) vs. asymptomatic patients living in South Italy and North-East Italy were 0.873 and 0.631 (p < 0.0001; p = 0.1116), respectively. AUC of patients with clinical severe reaction vs. all other reactions were 0.819 and 0.755 (p < 0.0001; p = 0.12), respectively.

The risk assessment of severe allergic reactions was the following: for a value of sIgE to Pru p 3 >7.5 kUA/L, the odds ratio 95 % CI was 69 (2.3–2028; p = 0.01) and >10 kUA/L, the odds ratio 95 % CI was 225 (3.9–13,114; p = 0.009).

## Discussion

For the first time, a study evaluates the usefulness of an anaphylactic biomarker in the risk assessment of Pru p 3 positive patients from different geographical areas of the same country.

In this study, we investigated the presence of correlations between the levels of sIgE to Pru p 3 with the severity of the clinical reactions in a South Italian population and compared them, with the results from a Nord-East Italian population, where the prevalence of sensitization to birch pollen is similar to North Europe.

All patients were assigned to one of the four groups according to their clinical manifestations: asymptomatic, OAS, mild systemic reactions and severe systemic reactions. We found that levels of sIgE to Pru p 3 (Mann–Whitney test) in the South population was significantly different between asymptomatic vs. symptomatic (OAS, mild and severe systemic reactions) Pru p 3 positive patients and between patients with mild systemic reactions in comparison to those with severe systemic reactions. Surprising, we could not found any significant differences in the levels of sIgE to Pru p 3 (Mann–Whitney test) in none of the groups from North-East Italy.

Few studies have assessed the relationship between the levels of sIgE to Pru p 3 and the severity of the clinical reactions, and taken all together provided contradictory results. Gaier et al. [26] have reported that the presence of sIgE to Pru p 3 in peach allergic patients who live in a geographical area with a high sensitization to birch pollen, may be considered a marker of severe reactions. On the other hand, Novembre et al. [21] have not found any relations between the levels of sIgE and the severity of the clinical reaction in a pediatric population; while Pastorello et al. [23] have found sIgE levels to Pru p 3 significantly higher in patients with severe OAS than in patients with mild OAS. In this study, we found a significant correlation between the severity of the clinical reaction and the levels of sIgE to Pru p 3. The differences in the results obtained could be attributed to the different type of population included in the studies.

An important result of this study was the determination of a cut-off level for sIgE to Pru p 3 values that can better discriminate asymptomatic patients from those symptomatic (2.87 kUA/L for the South Italian population and 2.69 kUA/L for the North-East population) and within this group, identify those patients at a higher risk for developing severe allergic reactions.

Pastorello et al. [23] have identified a cut-off value of sIgE to Pru p 3 of 2.69 kUA/L with a lower sensitivity 63.9 % but similar specificity 75 % that better discriminated peach-allergic patients at a higher risk for developing more severe symptoms.

Interestingly, we found a value of sIgE to Pru p 3 higher than 10.2 kUA/L (sensitivity almost of 90 %) that was present in 70 % of the patients with severe systemic reactions from South Italy. In the North-East group, with the same value of sIgE to Pru p 3, we have a higher specificity but a lower sensitivity. However, we cannot exclude that these results are probably affected by a bias due to the low numbers of patients with severe allergic reactions included in this population.

Although it has been shown that panallergens differ according to the geographical areas [4–6], up to now, nobody has studied the prevalence of peach panallergen in Sicily. The high sensitization to nsLTP and the lower prevalence of sensitization to profilin have been previously reported in a study done with Sicilian and Austrian patients allergic to Parietaria judaica [27]. In this regard, a study done in allergic patients (Uasuf et al. 2014 unpublished data) to assess the prevalence of panallergens in South Italy, has found that in 599 positive molecular ImmunoCAP tests performed, the prevalence of profilin was 3.6 % and of PR-10 was only 1 %.

The different clinical manifestations present in the two Italian populations could be attributed not only to the existence of different co-sensitization to the panallergens but also to the levels of sIgE to Pru p 3. Therefore, lower levels of sIgE to Pru p 3 and the co-existence of positivity to Pru p1 and/or Pru p 4 could explain the minor frequency of severe reactions in the North-East patients with respect to the South patients, monosensitized and with higher level of sIgE to Pru p 3. On a speculative level, this fact supports the hypothesis that patients positive to Pru p 3 and simultaneously positive to Pru p 1 and/or Pru p 4, have a sort of ‘protective’ effect against the development of severe symptoms induced by Pru p 3 [23].

## Conclusion

Our results show that the level of an anaphylactic biomarker like sIgE to Pru p 3, indicates the possibility of development a severe food allergic reaction. In addition, Pru p 3 positive patients from different geographical areas and with different co-sensitizations could have a different risk assessment in peach allergy.

This study could be a model to extrapolate to other geographical areas with different co-sensitizations to identify probably high risk group in food allergy.

Further studies, including multicentre studies are necessary to confirm and expand these results.

## Abbreviations

nsLTP: non specific lipid transfer protein
sBT: serum basal tryptase
FA: food allergy
sIgE: specific immunoglobulin E
CRD: component resolved diagnosis
PR-10: pathogenesis related protein-10
OAS: oral allergic syndrome
SPT: skin prick test

## References

[1] Sampson HA, Ho DG (1997). Relationship between food-specific IgE concentrations and the risk of positive food challenges in children and adolescents. J Allergy Clin Immunol..

[2] Yunginger JW, Ahlstedt S, Eggleston PA, Homburger HA, Nelson HS, Ownby DR, Platts-Mills TA, Sampson HA, Sicherer SH, Weinstein AM, Williams PB, Wood RA, Zeiger RS (2000). Quantitative IgE antibody assays in allergic diseases. J Allergy Clin Immunol..

[3] Sampson HA (2001). Utility of food-specific IgE concentrations in predicting symptomatic food allergy. J Allergy Clin Immunol..

[4] Asero R, Antonicelli L, Arena A, Bommarito L, Caruso B (2009). Causes of food-induced anaphylaxis in Italian adults: a multi-centre study. Int Arch Allergy Immunol.

[5] Fernandez-Rivas M, Bolhaar S, Gonzalez-Mancebo E, Asero R, van Leeuwen A (2006). Apple allergy across Europe: how allergen sensitization profiles determine the clinical expression of allergies to plant foods. J Allergy Clin Immunol.

[6] Zuidmeer L, van Ree R (2007). Lipid transfer protein allergy: primary food allergy or pollen/food syndrome in some cases. Curr Opin Allergy Clin Immunol.

[7] Egger M, Hauser M, Mari A, Ferreira F, Gadermaier G (2010). The role of lipid transfer proteins in allergic diseases. Curr Allergy Asthma Rep.

[8] Wijesinha-Bettoni R, Alexeev Y, Johnson P, Marsh J, Sancho AI (2010). The structural characteristics of nonspecific lipid transfer proteins explain their resistance to gastroduodenal proteolysis. Biochemistry.

[9] Asero R, Mistrello G, Roncarolo D, Amato S, Caldironi G, Barocci F, van Ree R (2002). Immunological cross-reactivity between lipid transfer proteins from botanically unrelated plant-derived foods: a clinical study. Allergy.

[10] Sánchez-Monge R, Lombardero M, García-Selles FJ, Barber D, Salcedo G (1999). Lipid transfer proteins are relevant allergens in fruit allergy. J Allergy Clin Immunol..

[11] Pastorello EA, Vieths S, Pravettoni V, Farioli L, Trambaioli C, Fortunato D, Lüttkopf D, Calamari M, Ansaloni R, Scibilia J, Ballmer-Weber BK, Poulsen LK, Wütrich B, Hansen KS, Robino AM, Ortolani C, Conti A (2002). Identification of hazelnut major allergens in sensitive patients with positive doubleblind, placebo-controlled food challenge results. J Allergy Clin Immunol..

[12] Asero R (2011). Lipid transfer protein cross-reactivity assessed in vivo and in vitro in the office: pros and cons. J Investig Allergol Clin Immunol.

[13] Pastorello EA, Farioli L, Pravettoni V, Ispano M, Scibola E, Trambaioli C, Giuffrida MG, Ansaloni R, Godovac-Zimmermann J, Conti A, Fortunato D, Ortolani C (2000). The maize major allergen, which is responsible for food-induced allergic reactions, is a lipid transfer protein. J Allergy Clin Immunol..

[14] Pastorello EA, Farioli L, Pravettoni V, Robino AM, Scibilia J, Fortunato D, Conti A, Borgonovo L, Bengtsson A, Ortolani C (2004). Lipid transfer protein and vicilin are important walnut allergens in patients not allergic to pollen. J Allergy Clin Immunol..

[15] Lauer I, Dueringer N, Pokoj S, Rehm S, Zoccatelli G, Reese G, Miguel-Moncin MS, Mistero Bahima A, Enrique E, Lidholm J, Vieths S, Scheurer S (2009). The non-specific lipid transfer protein, Ara h 9, is an important allergen in peanut. Clin Exp Allergy.

[16] Díaz Perales A, Lombardero M, Sánchez-Monge R, García-Selles FJ, Pernas M, Fernández-Rivas M, Barber D, Salcedo G (2000). Lipid-transfer proteins as potential plant panallergens: cross-reactivity among proteins of Artemisia pollen, Castanea nut and Rosaceae fruits, with different IgE-binding capacities. Clin Exp Allergy.

[17] Ciardiello MA, Palazzo P, Bernardi ML, Carratore V, Giangrieco I, Longo V, Melis M, Tamburrini M, Zennaro D, Mari A, Colombo P (2010). Biochemical, immunological and clinical characterization of a cross-reactive nonspecific lipid transfer protein 1 from mulberry. Allergy.

[18] Pastorello EA, Farioli L, Stafylaraki C, Scibilia J, Giuffrida MG, Mascheri A, Piantanida M, Baro C, Primavesi L, Nichelatti M, Schroeder JW, Pravettoni V (2013). Fennel allergy is a lipid-transfer protein (LTP)-related food hypersensitivity associated with peach allergy. J Agric Food Chem.

[19] Pastorello EA, Scibilia J, Farioli L, Primavesi L, Giuffrida MG, Mascheri A, Piantanida M, Mirone C, Stafylaraki C, Violetta MR, Nichelatti M, Preziosi D, Losappio L, Pravettoni V (2013). Rice allergy demonstrated by double-blind placebo-controlled food challenge in peach-allergic patients is related to lipid transfer protein reactivity. Int Arch Allergy Immunol.

[20] Asero R, Mistrello G, Roncarolo D, Amato S (2004). Relationship between peach lipid transfer protein specific IgE levels and hypersensitivity to non-Rosaceae vegetable foods in patients allergic to lipid transfer protein. Ann Allergy Asthma Immunol.

[21] Novembre E, Mori F, Contestabile S, Rossi ME, Pucci N (2012). Correlation of anti-Pru p 3 IgE levels with severity of peach allergy reactions in children. Ann Allergy Asthma Immunol.

[22] Pastorello EA, Farioli L, Stafylaraki C, Mascheri A, Scibilia J, Pravettoni V, Primavesi L, Piantanida M, Nichelatti M, Asero R (2013). Anti-rPru p 3 IgE levels are inversely related to the age at onset of peach-induced severe symptoms reported by peach-allergic adults. Int Arch Allergy Immunol.

[23] Pastorello EA, Farioli L, Pravettoni V, Scibilia J, Mascheri A, Borgonovo L, Piantanida M, Primavesi L, Stafylaraki C, Pasqualetti S, Schroeder J, Nichelatti M, Marocchi A (2011). Pru p 3-sensitised Italian peach-allergic patients are less likely to develop severe symptoms when also presenting IgE antibodies to Pru p 1 and Pru p 4. Int Arch Allergy Immunol..

[24] Mueller HL (1966). Diagnosis and treatment of insect sensitivity. J Asthma Res..

[25] Bindslev-Jensen C, Ballmer-Weber BK, Bengtsson U, Blanco C, Ebner C, Hourihane J, Knulst AC, Moneret-Vautrin DA, Nekam K, Niggemann B, Osterballe M, Ortolani C, Ring J, Schnopp C, Werfel T (2004). Standardization of food challenges in patients with immediate reactions to foods—position paper from the European Academy of Allergology and Clinical Immunology. Allergy.

[26] Gaier S, Oberhuber C, Hemmer W, Radauer C, Rigby NM, Marsh JT, Mills CEN, Shewry PR, Hoffmann-Sommergruber K (2009). Pru p 3 as a marker for symptom severity for patients with peach allergy in a birch pollen environment. J Allergy Clin Immunol.

[27] Stumvoll S, Westritschnig K, Lidholm J, Spitzauer S, Colombo P, Duro G, Kraft D, Geraci D, Valenta R (2003). Identification of cross-reactive and genuine *Parietaria judaica* pollen allergens. J Allergy Clin Immunol..

